# Supplementary material for: Variants Affecting Exon Skipping Contribute to Complex Traits
Source: PLoS Genet. 2012 Oct 25;8(10):e1002998. doi: 10.1371/journal.pgen.1002998 (PMC3486879; doi:10.1371/journal.pgen.1002998)
Supplement: Figure S1 — Enrichment of Exon Skipping–Associated ISE SNPs among Complex Human Trait-Associated SNPs. The distribution of the number of predicted exon-skipping associated ISE SNPs observed for each of the 1,000 random sets (each consisting of 3,353 SNPs, equal to the number of trait-associated SNPs) while conditioning on MAF (using HapMap CEU MAF data) and distance to the nearest exon is shown in the bar graphs. The actual number of 111 predicted exon-skipping ISE SNPs observed in the 3,353 SNPs from the NHGRI catalog is shown as a solid asterisk. (PDF) [file pgen.1002998.s001.pdf]

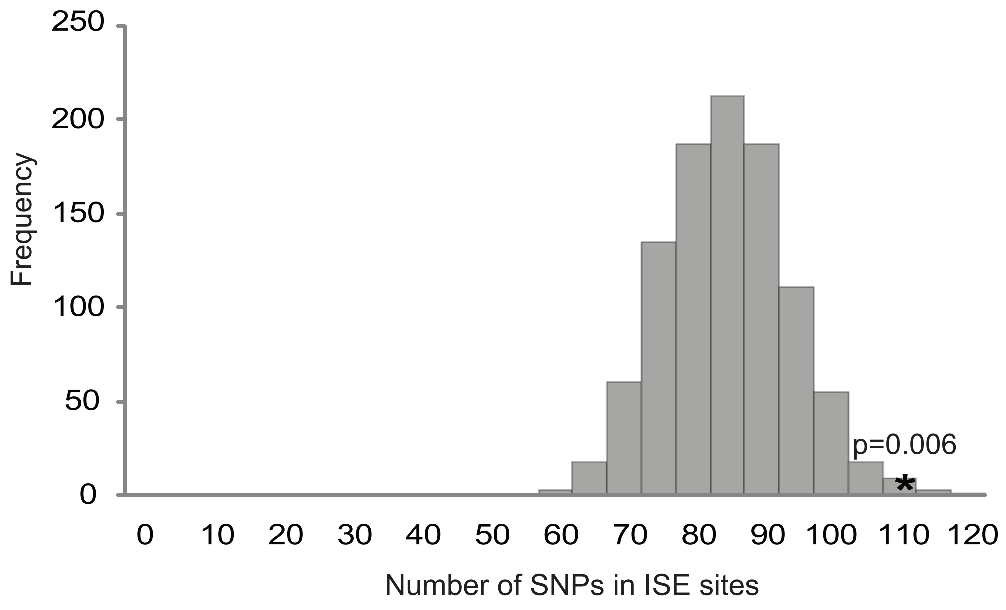

Figure S1. Enrichment of Exon Skipping-Associated ISE SNPs among Complex Human Trait-Associated SNPs. The distribution of the number of predicted exon-skipping associated ISE SNPs observed for each of the 1,000 random sets (each consisting of 3,353 SNPs, equal to the number of trait-associated SNPs) while conditioning on MAF (using HapMap CEU MAF data) and distance to the nearest exon is shown in the bar graphs. The actual number of 111 predicted exon-skipping ISE SNPs observed in the 3,353 SNPs from the NHGRI catalog is shown as a solid asterisk.
