# Supplementary material for: Variants Affecting Exon Skipping Contribute to Complex Traits
Source: PLoS Genet. 2012 Oct 25;8(10):e1002998. doi: 10.1371/journal.pgen.1002998 (PMC3486879; doi:10.1371/journal.pgen.1002998)
Supplement: Figure S2 — SNPs Located in Multiple ISE Motifs Are Not Associated with a Change in Exon Skipping Level. The y-axis is the absolute value of 1 minus SI (|1-SI|); here, a higher level of |1-SI| corresponds to a higher level of exon skipping. We tested the correlation of genotype with exon skipping. There was no statistical correlation found between each SNP and exon skipping. (PDF) [file pgen.1002998.s002.pdf]

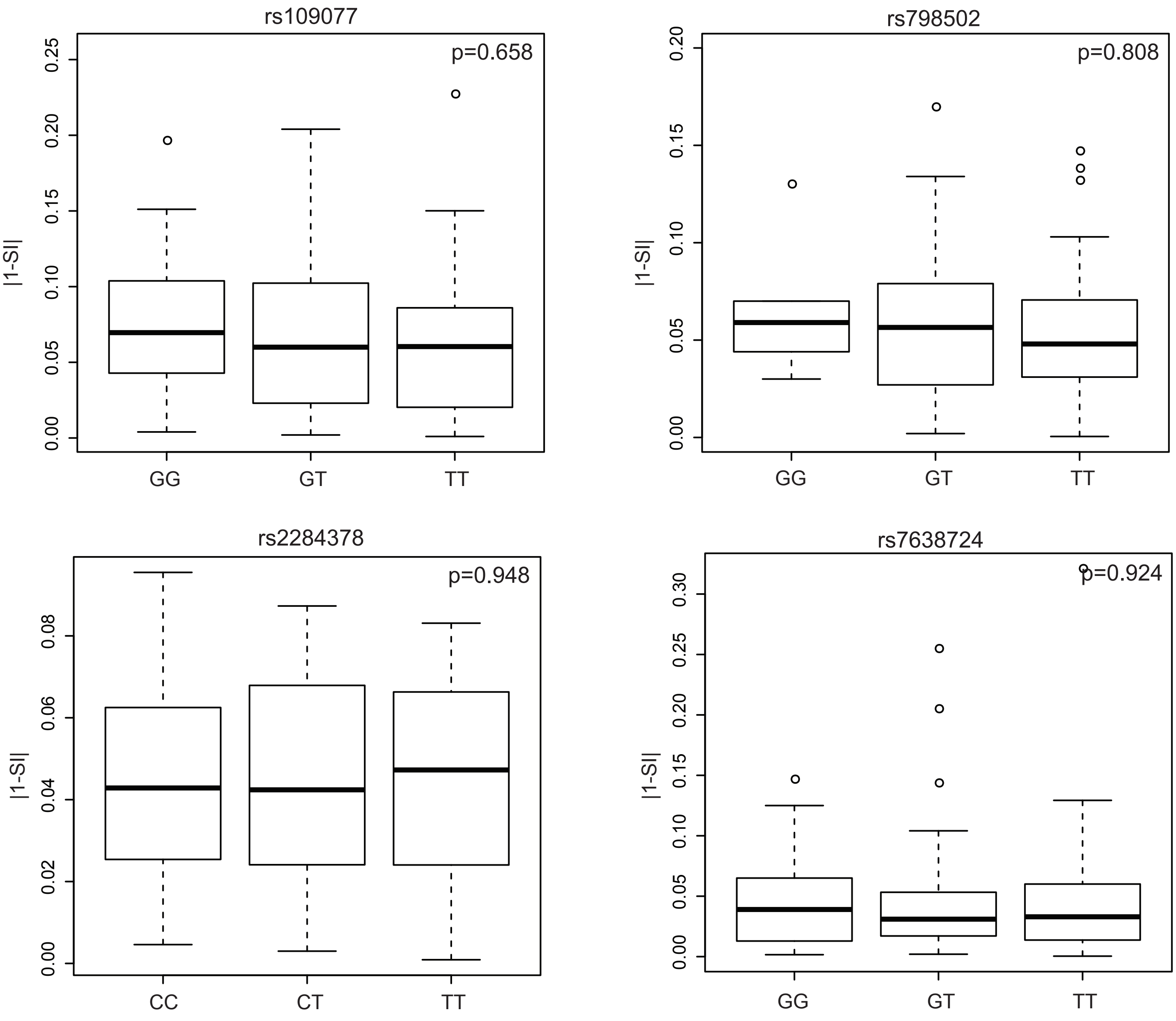

Figure S2. SNPs Located in Multiple ISE Motifs Are Not Associated with a Change in Exon Skipping Level. The y-axis is the absolute value of 1 minus SI (|1-SI|); here, a higher level of |1-SI| corresponds to a higher level of exon skipping. We tested the correlation of genotype with exon skipping. There was no statistical correlation found between each SNP and exon skipping.
