# Supplementary material for: Variants Affecting Exon Skipping Contribute to Complex Traits
Source: PLoS Genet. 2012 Oct 25;8(10):e1002998. doi: 10.1371/journal.pgen.1002998 (PMC3486879; doi:10.1371/journal.pgen.1002998)
Supplement: Figure S3 — ISE SNPs Are Not Likely to Be Closer to the Skipped Exon Than Intronic SNPs Are to Nearest Exon. P-value was calculated by using a two-sample Kolmogorov-Smirnov Test. (PDF) [file pgen.1002998.s003.pdf]

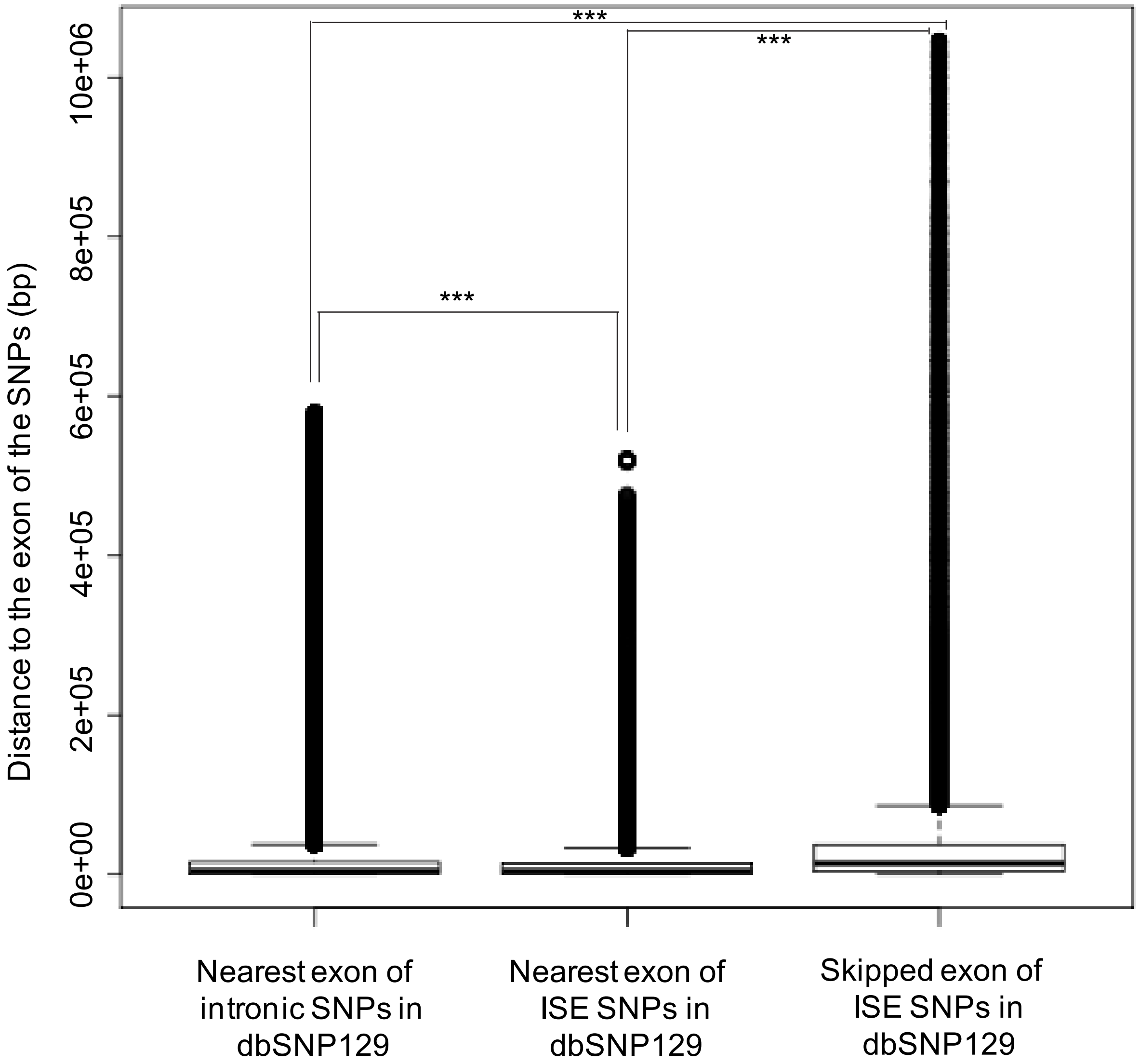

Figure S3. ISE SNPs Are Not Likely to Be Closer to the Skipped Exon Than Intronic SNPs Are to Nearest Exon. P-value was calculated by using a two-sample Kolmogorov-Smirnov Test.
