# Supplementary material for: Variants Affecting Exon Skipping Contribute to Complex Traits
Source: PLoS Genet. 2012 Oct 25;8(10):e1002998. doi: 10.1371/journal.pgen.1002998 (PMC3486879; doi:10.1371/journal.pgen.1002998)
Supplement: Figure S4 — ISE SNP (rs12924138) in CDK10. (A) Gene structures and transcript isoforms are annotated as in RefSeq Genes and Ensembl Gene Predictions archive 54. The second exon was found to be skipped. The SNP rs129224138 was predicted to be at the fifth site in AGCCTG ISE motif sequence and to be associated with the second exon's skipping. (B) The substitution T>G change the cis-acting sequence into a non cis-acting sequence. (C) Exon skipping level and splicing index ratio of the genotypes in the 176 HapMap samples. (PDF) [file pgen.1002998.s004.pdf]

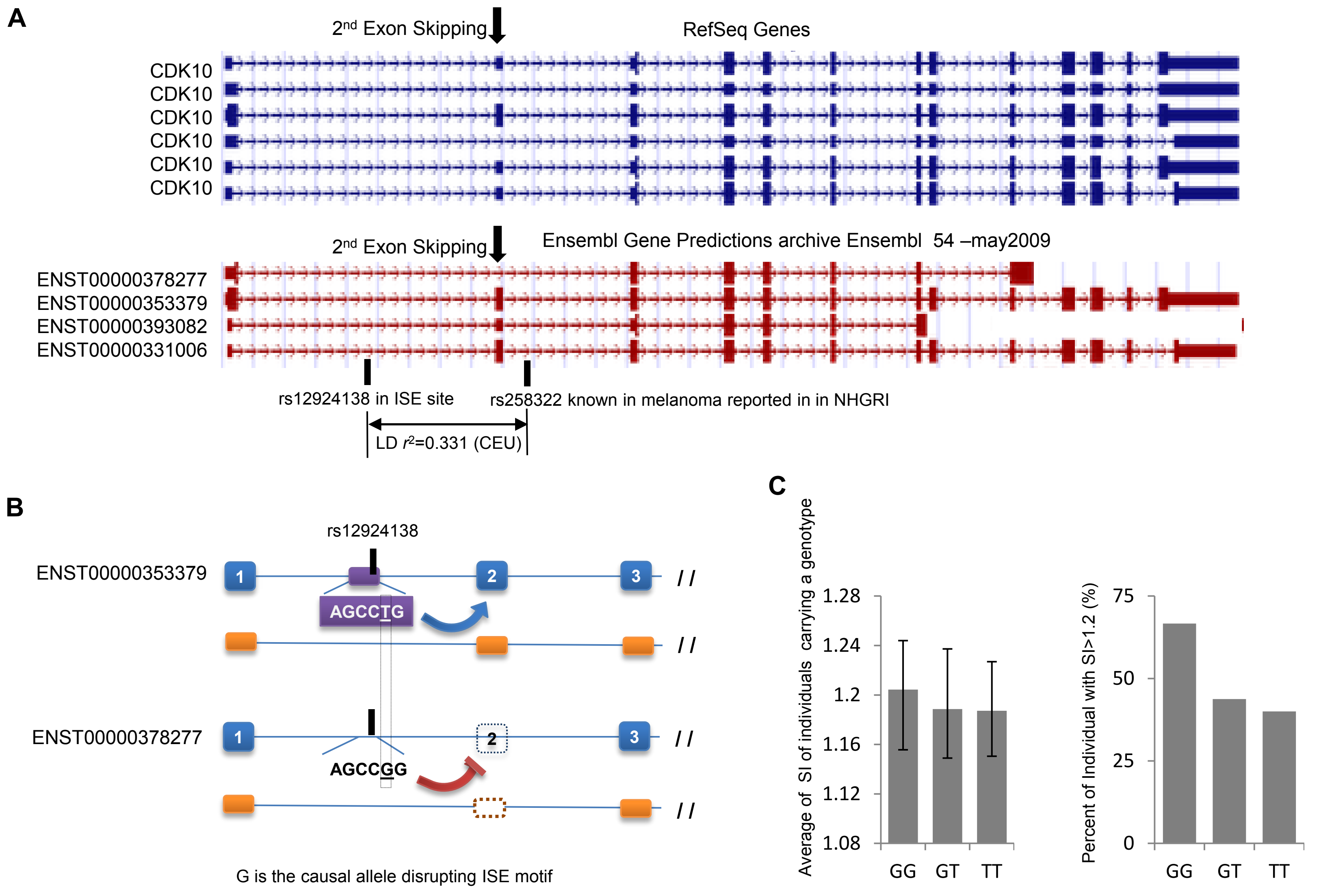

Figure S4. ISE SNP (rs12924138) in *CDK10*. (**A**) Gene structures and transcript isoforms are annotated as in RefSeq Genes and Ensembl Gene Predictions archive 54. The second exon was found to be skipped. The SNP rs129224138 was predicted to be at the fifth site in AGCCTG ISE motif sequence and to be associated with the second exon's skipping. (**B**) The substitution T>G change the cis-acting sequence into a non cis-acting sequence. (**C**) Exon skipping level and splicing index ratio of the genotypes in the 176 HapMap samples.
