# Supplementary material for: Variants Affecting Exon Skipping Contribute to Complex Traits
Source: PLoS Genet. 2012 Oct 25;8(10):e1002998. doi: 10.1371/journal.pgen.1002998 (PMC3486879; doi:10.1371/journal.pgen.1002998)
Supplement: Table S1 — Enrichment of ISE SNPs for Human Trait-Associated SNPs. (PDF) [file pgen.1002998.s005.pdf]

Table S1. Enrichment of ISE SNPs for Human Trait-Associated SNPs.

Distance to splice junction of ISE SNP	Enrichment p-value when conditioning on MAF	Enrichment p-value when conditioning on both MAF and the distance to the nearest exon
<60bp	0.054	0.090
<200bp	0.004	0.032
<1kbp	0.033	0.340
<5kbp	0.015	0.279
All Distance	0.006	0.036
