# Supplementary material for: Variants Affecting Exon Skipping Contribute to Complex Traits
Source: PLoS Genet. 2012 Oct 25;8(10):e1002998. doi: 10.1371/journal.pgen.1002998 (PMC3486879; doi:10.1371/journal.pgen.1002998)
Supplement: Table S2 — Structural comparisons of the Selected Four Pairs of Alternative Splice Isoforms in Table 1. (PDF) [file pgen.1002998.s006.pdf]

Table S2. Structural Comparisons of the Selected Four Pairs of Alternative Splice Isoforms in Table 1.

Exon gene	Skipped exon[a]	Longer isoform[b]	Shorter isoform[c]	C-score[d]	RMSD (Å)[e]	TM-score[f]
RNF8	7 (69 aa)	ENSP00000362578	ENSP00000229866	-1.28/-2.30	7.34	0.199
SLC25A15	3 (46 aa)	ENSP00000342267	ENSP00000368838	1.39/-0.81	3.57	0.354
PPP5C	5 (22 aa)	ENSP00000012443	ENSP00000375786	0.22/0.36	0.66	0.739
NARF	3 (45 aa)	ENSP00000309899	ENSP00000283996	0.38/1.00	2.37	0.809

[a] Position of skipped exon in longer isoform. The length of amino acid encoded by the skipped exon is given in parenthesis.

[b] Isoform including the exon which is skipped in shorter isoform.

[c] Isoform in which exon is skipped.

[d] Confidence score for the quality of predicted models by I-TASSER. C-score of higher value signifies a model with a high confidence and vice-versa. (longer isoform/shorter isoform)

[e] Value between residues that are structurally aligned by TM-align.

[f] Scale for measuring the structural similarity between two structures. TM-score >0.5 indicates a model of correct topology and a TM-score<0.17 means a random similarity.
